# Safety, Feasibility, and Exploratory Functional Changes During GRILLO© Gait Trainer Use in Adults with Severe Acquired Brain Injury: A Retrospective Observational Study

**DOI:** 10.3390/brainsci16060631

**Published:** 2026-06-12

**Authors:** Donatella Saviola, Stefania Bruni, Andrea Rattotti, Raffaella Benoldi, Katia Cristella, Elisa Quintavalla, Monica Pizzaferri, Antonio De Tanti

**Affiliations:** Centro Cardinal Ferrari KOS, 43012 Fontanellato, Italy; donatella.saviola@kosgroup.com (D.S.); andrea.rattotti@gmail.com (A.R.); raffaellabeno@libero.it (R.B.); katia.cristella77@gmail.com (K.C.); elisaquintavalla@yahoo.it (E.Q.); monica.pizzaferri@kosgroup.com (M.P.); antonio.detanti@kosgroup.com (A.D.T.)

**Keywords:** gait trainer, assisted verticalization, motor impairment, inpatient rehabilitation, trunk control, ambulation, severe acquired brain injury (sABI), traumatic brain injury (TBI)

## Abstract

**Highlights:**

**What are the main findings?**

- GRILLO-based training could be implemented in selected adults with severe acquired brain injury within routine inpatient multidisciplinary rehabilitation.
- No serious device-related adverse events were documented in available clinical records; however, adverse events were not monitored prospectively or systematically.
- Exploratory pre–post changes were observed in trunk control, balance, and activities of daily living, with substantial individual variability and floor effects in balance and gait-related measures.

**What are the implications of the main findings?**

- GRILLO may represent a useful therapeutic option for assisted verticalization and early task-oriented upright activity in selected patients who cannot yet access less-supported gait interventions.
- Feasibility, safety, and device-specific efficacy require prospective controlled studies with predefined feasibility endpoints, standardized safety monitoring, clearer patient selection criteria, and more sensitive outcome measures.

**Abstract:**

**Background/Objectives:** Assisted verticalization and supported upright activity are relevant components of rehabilitation in adults with severe acquired brain injury (sABI), although patient selection and implementation remain challenging. This retrospective observational study aimed primarily to describe the implementation feasibility and documented safety of GRILLO-based training in routine inpatient multidisciplinary rehabilitation, and secondarily to report exploratory pre–post functional changes. **Methods:** We reviewed clinical records of 34 adults screened or considered for GRILLO-based training at Centro Cardinal Ferrari KOS, Italy, between June 2022 and December 2024. GRILLO training was delivered as part of standard care and not as an experimental intervention. Functional outcomes included the Barthel Index (BI), Trunk Control Test (TCT), Tinetti Balance Scale, and Tinetti Gait subscale, extracted from routine documentation. Non-parametric descriptive analyses were used. **Results:** Of 34 screened patients, 4 did not meet diagnostic criteria for ABI, 5 interrupted training because of pain or poor tolerance to prolonged upright positioning, and 3 were not included because of poor compliance/motivation or an incomplete clinical pathway. The paired functional-analysis cohort comprised 22 patients: 20 (91%) completed 15 sessions and 2 (9%) completed 10 sessions. No serious device-related adverse events were documented in available clinical records, although minor adverse events were not systematically monitored. Among patients with paired observations, median BI increased from 16 to 22.5 (median change, +3; *p* = 0.008; n = 20), median TCT from 72 to 74 (median change, +12; *p* < 0.001; n = 21), and median Tinetti Balance Scale from 1 to 2 (median change, +1; *p* = 0.006; n = 22). Individual responses were heterogeneous and floor effects were evident, especially for balance and gait-related measures. **Conclusions:** In this retrospective real-world cohort, GRILLO-based training could be implemented in selected severely impaired inpatients, but feasibility may be overestimated if interrupted and non-completing cases are not considered. The non-completion cases may suggest that feasibility depends not only on initial clinical indication, but also on the appropriate timing of introduction, tolerance to prolonged upright physical effort, pain/discomfort, motivation, and behavioral engagement. The retrospective design, survivorship bias, non-systematic adverse-event monitoring, concurrent multidisciplinary rehabilitation, and absence of a comparator group preclude conclusions regarding device-specific safety or efficacy. Nevertheless, these preliminary findings support further prospective controlled studies.

## 1. Introduction

Acquired brain injury (ABI), including stroke, traumatic brain injury (TBI), anoxic injury, and other non-progressive neurological conditions [1], is a major cause of long-term disability in adults [2,3,4]. Traumatic brain injury (TBI) alone accounts for a substantial and growing global burden [5]. Across this spectrum, severe motor impairment remains one of the most disabling sequelae, limiting transfers, standing, walking, and participation in daily activities [6]. In patients with marked weakness, bilateral motor involvement, ataxia, or poor trunk control, one of the first rehabilitation challenges is not gait training itself, but the ability to achieve and tolerate safe upright positioning.

Assisted verticalization is a relevant component of contemporary rehabilitation practice [7,8]. Earlier and more intensive mobilization may support functional recovery and reduce immobilization-related complications in selected neurological populations [9,10]. However, evidence is not uniform across all severe neurological presentations, and mobilization must be adapted to medical stability, orthostatic tolerance, cognitive–behavioral profile, postural control, and safety considerations. The proposed mechanisms underlying potential benefits include preservation of muscle mass, prevention of learned nonuse, enhancement of neuroplasticity through task-specific practice, and maintenance of cardiovascular conditioning [9,11].

However, conventional approaches do not always address this need adequately. Standard standing systems may provide stability but often allow limited dynamic activity, whereas treadmill-based or robotic gait devices usually require a degree of postural control, upright tolerance, and organizational support that is not consistently available in severely impaired inpatients [12,13,14]. In clinical practice, some patients therefore fall into a functional “therapeutic grey zone”: they are too impaired for conventional gait training but may still be able to participate in task-oriented upright activity when adequate stabilization, partial unloading, and therapist assistance are provided. In the present study, this subgroup was operationally represented by patients with severe limitations in standing and walking for whom the treating multidisciplinary team prescribed GRILLO-based training as part of routine care.

This gap has increased interest in modular assistive devices capable of combining safety with active participation [7,8,15]. For this population, an appropriate device should not simply maintain the patient in an upright position. It should allow individualized adjustment of pelvic and trunk support, partial unloading when necessary, and progressive reduction of assistance as postural control and tolerance improve. These features are particularly relevant in patients with fluctuating trunk stability, bilateral weakness, cerebellar signs, or mixed motor presentations. In addition, clinical usefulness is influenced not only by biomechanical design, but also by practical factors such as ease of set-up, therapist workload, acceptance by patients and caregivers, and the possibility of transfer from supervised clinical use to everyday environments [11].

The Gait Trainer GRILLO was introduced in our inpatient rehabilitation setting for severe acquired brain injury as a modular gait trainer for patients with severe motor impairment who were unable to access less-supported gait interventions. In clinical use, the device was primarily employed to facilitate assisted verticalization, trunk control, weight shifting, and gait-related preparation in patients with limited standing and walking abilities. The rationale for using GRILLO was based on its modular support system, which allows trunk and pelvic stabilization, graded assistance, and progressive adaptation of support according to patient tolerance and motor control. However, feasibility in the rehabilitation gym does not automatically imply suitability for home use, and patient selection remains a key issue.

The present retrospective study was designed to describe the use of the Gait Trainer GRILLO in a real-world inpatient rehabilitation population. The primary objectives were to describe implementation feasibility and documented safety in routine clinical practice. Secondary objectives were to report exploratory pre–post functional changes observed during the period in which GRILLO-based training was delivered in addition to standard multidisciplinary rehabilitation. Because of the retrospective design, diagnostic and chronicity heterogeneity, concurrent rehabilitation, and absence of a comparator group, the study was not intended to determine device-specific efficacy. Rather, it was conceived as a preliminary clinical description to clarify whether this approach could be implemented in routine practice, which patients accessed or did not complete it, and whether the available data justified further controlled investigation.

## 2. Materials and Methods

### 2.1. Study Design

This retrospective observational study was conducted at Centro Cardinal Ferrari KOS, an inpatient rehabilitation facility in Italy. We reviewed medical records of patients who had received or had been considered for GRILLO-based training as part of routine clinical care between June 2022 and December 2024. The primary aim was to describe implementation feasibility and documented safety; exploratory functional changes were analyzed only in patients with paired outcome data. The study is reported in accordance with STROBE recommendations for observational studies [16,17].

All GRILLO training sessions were delivered as part of standard inpatient multidisciplinary rehabilitation, not as a research protocol. Functional assessments were performed as part of routine clinical documentation at admission, during rehabilitation, and at discharge, and were retrospectively extracted from clinical records for the purposes of this study. The retrospective study protocol was reviewed and authorized by the Scientific Board of KOS on 14 January 2026, before data extraction began, under protocol code 01/2026. All patients or their legal representatives had provided general consent for use of anonymized clinical data for research purposes at the time of admission, as per institutional policy. Separate written informed consent for publication was obtained for any patient images included in the manuscript.

### 2.2. Patient Selection and Data Extraction

The study population included patients with acquired neurological conditions associated with severe limitations in standing and walking, including TBI, stroke, anoxic injury, hemorrhagic lesions, and other non-progressive disorders affecting postural control and mobility.

Patients were retrospectively identified through the clinical database. Eligibility for GRILLO-based training was determined clinically by the treating multidisciplinary team and was limited to adults admitted for inpatient multidisciplinary rehabilitation after sABI with neurological motor impairment and severe limitations in standing or walking. For feasibility reporting, all screened or considered patients were described, including patients who did not start or did not complete the planned training. For exploratory functional analyses, the paired functional-analysis cohort included patients who completed at least ten GRILLO training sessions and had functional assessments available both at baseline and after the intervention period. This minimum exposure criterion was defined retrospectively and may have introduced survivorship bias. Patients were not included in the paired functional analysis if they completed fewer than ten sessions, if training was stopped within the first sessions because of poor tolerance or emerging medical conditions, if documentation was incomplete, or if discharge occurred before reassessment. Further exclusion criteria to GRILLO training were reconstructed from routine clinical documentation. In practice, these included persistent orthostatic hypotension despite treatment, severe diffuse hypotonia precluding safe positioning, very severe cognitive–behavioral impairment (Level of Cognitive Functioning < 6), absence of postural support reactions, unstable medical conditions incompatible with upright activity, major orthopedic restrictions such as unconsolidated skeletal lesions, and anthropometric incompatibility with the device.

### 2.3. Device Description

**Figure 1:**
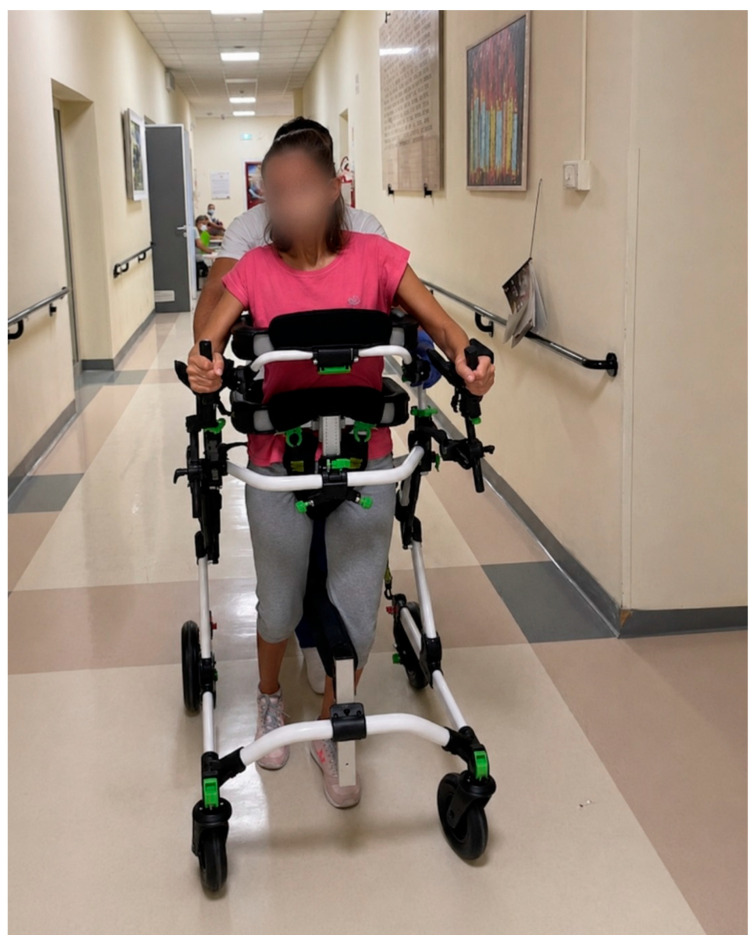
Gait Trainer GRILLO (Ormesa, Italy). The device is shown with its modular configuration, including trunk and pelvic supports, harness system, and adjustable frame. Components can be adapted according to patient-specific motor and postural needs.

The Gait Trainer GRILLO (Ormesa S.r.l, Foligno PG, Italy) is a modular gait trainer designed to support assisted verticalization and gait-related training in individuals with motor impairment. The device is based on a lightweight frame and can be configured in anterior or posterior versions according to the user’s postural and motor needs. In the clinical setting examined in this study, its main relevance was the possibility of combining upright support with active postural engagement in patients who could not yet access conventional gait training. An overview of the GRILLO device, including its main structural components and support modules, is shown in Figure 1.

The system allows independent adjustment of pelvic and trunk supports and can be equipped, according to patient needs, with an ergonomic harness, forearm supports, handles, saddle, abduction components, lower-limb separation guides, and wheel-control options. In practical terms, these components were used to modulate stabilization, partial unloading, and movement guidance during therapy. In selected patients with ataxic features, weighted bars were added as part of the configuration strategy. Device configuration was individualized according to residual motor control, tolerance to upright posture, and treatment goals, with progressive reduction of support when clinically feasible.

### 2.4. Intervention Description

**Figure 2:**
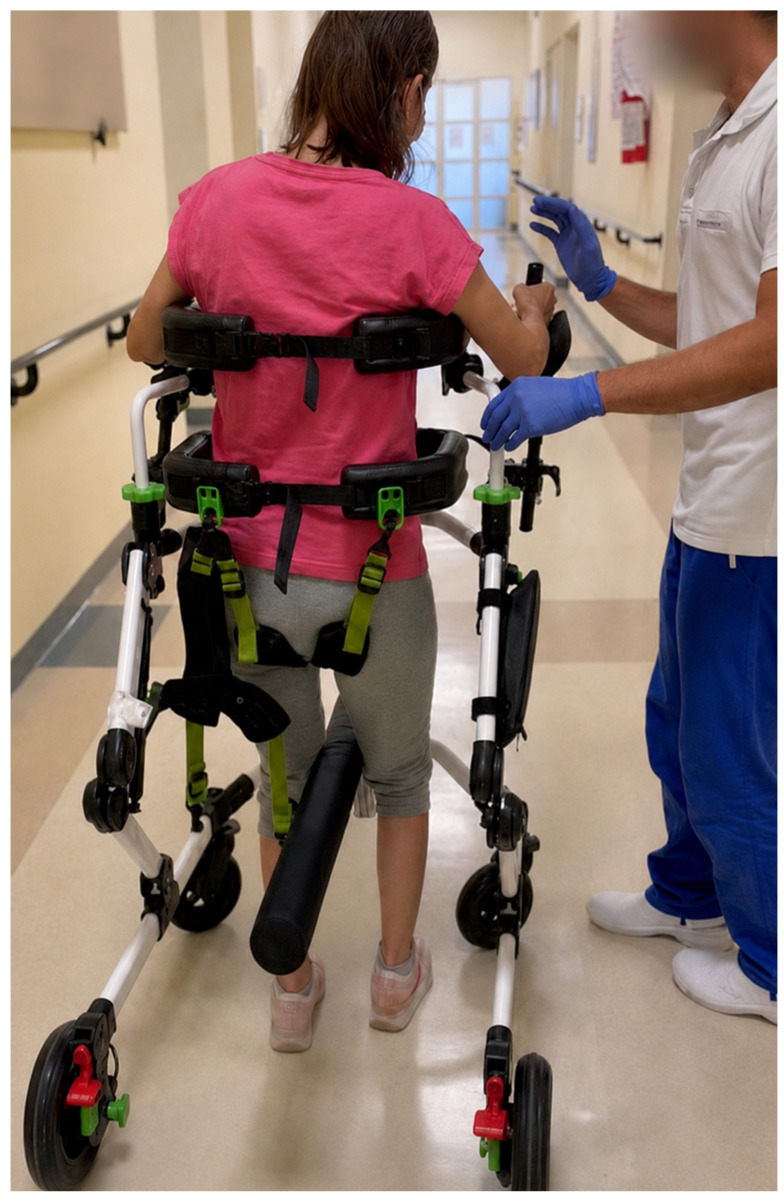
Representative example of GRILLO-assisted training in a patient with severe motor impairment. The device provides trunk and pelvic support, enabling assisted verticalization and task-oriented activity under therapist supervision.

GRILLO-based training was delivered by experienced physiotherapists as part of the standard multidisciplinary rehabilitation program. Although individualized to patient needs, training followed a broadly consistent structure: initial sessions focused on safe positioning, assessment of tolerance to upright posture, and determination of appropriate device configuration. Subsequent sessions addressed assisted verticalization, static and dynamic trunk control, weight shifting, postural alignment, stepping preparation, and, when feasible, supported gait-related activity. Progression was guided clinically by tolerance to upright posture, ability to maintain trunk and pelvic alignment, emergence of support reactions, ability to participate in weight shifting or stepping preparation, fatigue, discomfort, and safety considerations. The level of external support was progressively modified according to clinical response. A representative example of GRILLO-assisted training during a therapy session is shown in Figure 2.

The target duration was 15 sessions, typically delivered on weekdays within the regular rehabilitation schedule (3–4 sessions per week), with each session lasting approximately 30–45 min. Actual session frequency, duration, and number of sessions varied according to clinical course, tolerance, staffing requirements, and discharge timing. Because this was a retrospective real-world study, training dose was not experimentally controlled and no formal dose–response analysis was planned. Initial sessions focused on safe positioning, patient familiarization, and tolerance assessment. Device configuration (anterior/posterior orientation, harness type, support level, additional components) was determined individually by the treating physiotherapist based on patient motor profile and safety requirements.

All sessions were supervised by physiotherapists experienced in the management of severely impaired neurological patients. The level of staffing required for positioning and training varied according to baseline motor impairment and trunk control. Patients continued to receive standard multidisciplinary inpatient rehabilitation concurrent with GRILLO training, including physical therapy and, where clinically indicated, occupational therapy, neuropsychological intervention, and speech therapy. Because standard therapy exposure was not recorded in a sufficiently structured way for all patients, the independent contribution of GRILLO training could not be isolated.

### 2.5. Clinical Measures and Data Extraction

Clinical outcome measures were retrospectively extracted from medical records. Assessments had been performed by trained clinical staff as part of routine rehabilitation documentation, typically before the start of GRILLO training (baseline) and at completion of the training course or at discharge (post-intervention). When multiple assessments were available during the rehabilitation stay, the measurement closest to the beginning and end of the GRILLO training period was selected.

Functional independence was described using the Barthel Index (BI) [18,19], a widely used measure of performance in activities of daily living, with scores ranging from 0 to 100 and higher values indicating greater independence. Trunk function was assessed with the Trunk Control Test (TCT) [20,21], which evaluates rolling, sitting up from supine, and sitting balance, again on a 0–100 scale, with higher scores reflecting better trunk control. Balance and gait were documented using the Tinetti Performance-Oriented Mobility Assessment (POMA) [22,23]. The Tinetti Balance Scale ranges from 0 to 16, and the Tinetti Gait subscale ranges from 0 to 12; for both scales, higher scores indicate better performance. Given the severity of impairment in this cohort, both balance and gait subscores were extracted when available and were expected to show substantial floor effects, because many patients were unable to perform standing balance or stepping tasks at baseline. Walking speed was recorded only in those patients for whom overground ambulation became measurable during the treatment period [24].

In addition to functional outcomes, we extracted information on treatment completion, reasons for non-completion when documented, documented adverse events, and patient-reported perceptions of device usefulness when these were available in the clinical notes. Patient perceptions were not collected with a validated satisfaction or usability questionnaire and are therefore reported only descriptively.

### 2.6. Statistical Analysis

Due to the exploratory nature of the study, the retrospective design, and the marked heterogeneity of the cohort, the statistical analysis was intentionally descriptive and non-parametric. Continuous variables are reported as median and interquartile range (IQR), while categorical variables are presented as counts and percentages.

Pre–post comparisons were performed using the Wilcoxon signed-rank test on paired observations. Because denominators differed across outcomes according to data availability and assessment completion, the sample size for each comparison is reported explicitly. Effect size r was calculated as |z|/sqrt(n). Given the small, heterogeneous sample and the exploratory non-parametric design, confidence intervals for paired median changes were not estimated because they would be unstable and potentially misleading; change-score medians, IQRs, and effect sizes are reported to support interpretation of magnitude and dispersion. Statistical significance was set at *p* < 0.05. No correction for multiple comparisons was applied given the exploratory nature of the analysis.

Reference thresholds for clinically meaningful change were considered only as approximate interpretive anchors and not as definitive response criteria, particularly because available benchmarks are largely derived from more homogeneous neurological populations than the one included here.

The time elapsed between the neurological event and the start of GRILLO training was also described, as chronicity was expected to vary substantially across participants and could influence both tolerance and response to the intervention. Because the study was not powered for subgroup comparisons, no formal inferential subgroup analysis by diagnosis or chronicity was planned. Where subgroup or distributional information is presented, it should be interpreted as descriptive only.

## 3. Results

### 3.1. Patient Characteristics

**Figure 3:**
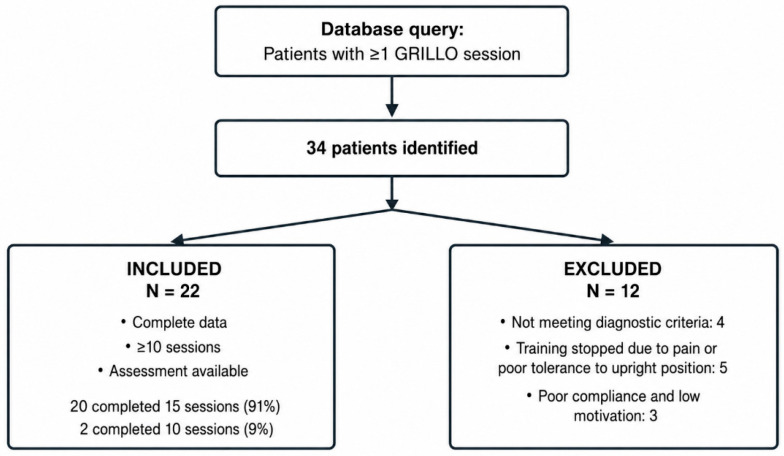
Patient selection and data availability flowchart. Flowchart of patient identification, exclusion, and inclusion. A total of 34 patients were identified through review of clinical records. Twelve were excluded: four did not meet diagnostic criteria (diagnosis not consistent with ABI), five had training interrupted due to pain or poor tolerance to prolonged upright position, and three showed poor compliance and low motivation. The final analysis cohort included 22 patients. Paired data were available for 20 patients for the Barthel Index, 21 for the Trunk Control Test, and 22 for the Tinetti Balance Scale.

A total of 34 patients were screened or considered for GRILLO-based training. The process of patient identification, exclusion, non-completion, and inclusion in the paired functional-analysis cohort is summarized in Figure 3. Four patients did not meet diagnostic criteria for ABI, five interrupted training because of pain or poor tolerance to prolonged upright positioning, and three were not included because of poor compliance/motivation or an incomplete clinical pathway. The final paired functional-analysis cohort included 22 patients with severe neurological motor impairment undergoing inpatient rehabilitation. The median age was 53 years (IQR 39–58, range 20–75), and 45% of participants were female.

Diagnoses included traumatic brain injury (n = 8), hemorrhagic stroke or subarachnoid hemorrhage (n = 6), ischemic stroke (n = 3), anoxic encephalopathy (n = 2), and other acquired neurological conditions (n = 3).

The median time from neurological event to the start of GRILLO training was 196 days (IQR 116–360, range 41–1888 days), indicating a population distributed across subacute (≤6 months: 45%) and chronic (>6 months: 55%) phases of recovery. The range includes one patient with an interval of approximately 4 years, reflecting the inclusion of individuals with long-standing but functionally relevant motor impairments. Most patients completed the planned training protocol, with 20 (91%) completing 15 sessions and 2 (9%) completing 10 sessions. Baseline demographic and clinical characteristics of the final study cohort are reported in Table 1.

### 3.2. Safety and Feasibility

Review of available clinical records revealed no serious adverse events attributed to device use. However, adverse events were not monitored prospectively or systematically, and minor events such as transient orthostatic symptoms, discomfort during positioning, fatigue, or pain may have been underreported. Therefore, safety findings should be interpreted as the absence of serious device-related events documented in routine records rather than as definitive evidence of device safety.

Among the 30 patients who met diagnostic eligibility for ABI and were considered potentially eligible for GRILLO-based training, 22 completed at least 10 sessions and had paired functional assessments. Thus, the completion rate for the paired functional-analysis cohort was 73% (22/30), whereas 8 of 30 patients (27%) did not complete the pathway or were not included in the paired analysis because of pain/poor tolerance, poor compliance/motivation, or an incomplete clinical pathway. Among the 22 patients included in the paired functional-analysis cohort, 20 (91%) completed 15 sessions and 2 (9%) completed 10 sessions. These findings support feasibility in selected patients but also indicate that feasibility would be overestimated if interpreted only among those who completed the treatment.

From a practical perspective noted in clinical documentation, the use of the device required variable levels of therapist assistance depending on baseline motor impairment. In more severely affected patients, positioning and initial sessions required substantial support, whereas in others the level of assistance decreased over the training period.

### 3.3. Functional Outcomes

Paired functional outcomes were available for a subset of patients depending on data completeness. Overall, pre–post comparisons showed exploratory improvements across measures of functional independence, trunk control, and balance, although individual responses varied substantially and causal attribution to GRILLO cannot be made because all patients received concurrent multidisciplinary rehabilitation and no comparator group was available. Overall pre–post changes in functional outcomes are summarized in Table 2.

Baseline, post-intervention, and change values are medians (IQRs) for paired observations. *p* values were obtained using the Wilcoxon signed-rank test. Effect size r was calculated as |z|/sqrt(n) and interpreted descriptively; no correction for multiple comparisons was applied. The Tinetti Gait subscale is reported descriptively only because of marked floor effects and limited score distribution; therefore, no inferential test or effect size was calculated. Confidence intervals for paired median changes were not estimated because of the small, heterogeneous sample and exploratory non-parametric design.

**Figure 4:**
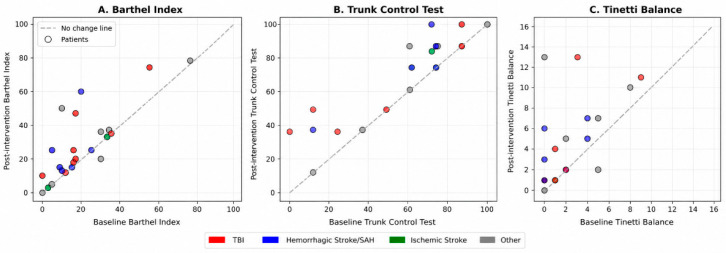
Individual pre- and post-intervention trajectories for functional outcomes. Scatter plots showing baseline and post-intervention scores for the Barthel Index (**A**), Trunk Control Test (**B**), and Tinetti Balance Scale (**C**). Each point represents an individual patient. Values above the dashed line represent improvement. Colors indicate diagnostic category.

Individual trajectories showed marked variability across patients. Some participants demonstrated clinically appreciable gains, whereas others remained stable or declined over the intervention period. This variability was observed across the main functional measures and is illustrated in Figure 4.

**Figure 5:**
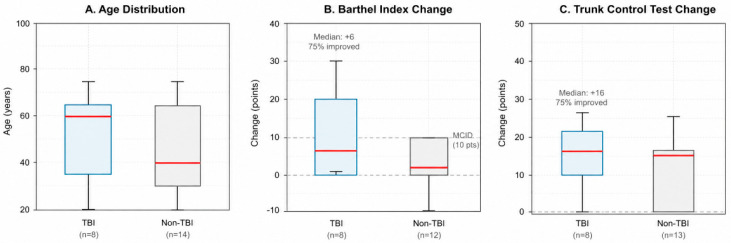
Descriptive analysis of the traumatic brain injury subgroup. Panel (**A**) shows the age distribution in patients with traumatic brain injury (TBI) and in the remainder of the cohort. Panels (**B**,**C**) show change scores for the Barthel Index and Trunk Control Test, respectively. Red horizontal lines indicate median values. In Panel B, the dashed horizontal line indicates an approximate minimal clinically important difference (MCID) reference threshold of 10 points for the Barthel Index; this threshold is used only as an interpretive anchor and not as a formal response criterion in this heterogeneous cohort. No formal statistical comparison between subgroups was performed because of the limited sample size.

A descriptive subgroup analysis was also performed for patients with traumatic brain injury (see Figure 5), given that this represented one of the largest diagnostic groups in the cohort.

**Figure 6:**
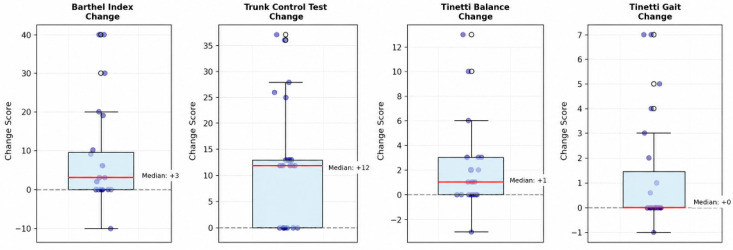
Distribution of change scores. Box plots showing the distribution of change scores for the Barthel Index, Trunk Control Test, Tinetti Balance Scale, and Tinetti Gait subscale. Circles represent individual patient data points are superimposed on each box plot. The red horizontal line indicates the median value. The dashed line represents zero change (no improvement or decline).

The intended purpose of this figure is descriptive only: it illustrates heterogeneity within and outside the TBI subgroup and does not imply a formal comparison between diagnostic groups. The distribution of change scores, as shown in Figure 6, further confirmed this variability. Rather than showing a uniform pattern of response, post-intervention changes were dispersed across a broad range, indicating different degrees of functional change at the individual level.

#### 3.3.1. Barthel Index

Paired observations were available for 20 patients. Median BI increased from 16 (IQR 9.75–26.25) at baseline to 22.5 (IQR 14.5–35.5) post-intervention, corresponding to a median change of +3 points (IQR 0–12.25). The Wilcoxon signed-rank test indicated a statistically significant difference (*p* = 0.008; effect size r = 0.59). Individual trajectories showed marked variability. Among the patients with paired data, 12 (60%) demonstrated improvement, 7 (35%) remained stable, and 1 (5%) showed decline. The median change was below commonly cited thresholds for clinically meaningful change in stroke populations, and therefore the result should be interpreted as a small group-level shift with heterogeneous individual responses rather than as clear evidence of functional efficacy.

#### 3.3.2. Trunk Control Test

Paired observations were available for 21 patients. Median TCT scores increased from 72 (IQR 37–74) at baseline to 74 (IQR 49–87) post-intervention, with a median change of +12 points (IQR 0–13). This change was statistically significant (*p* < 0.001; effect size r = 0.77). Among these patients, 15 (71%) improved, 6 (29%) remained stable, and none showed decline. Compared with other outcomes, improvements in trunk control appeared more consistent across patients, although variability remained substantial. However, interpretation remains exploratory because improvements may reflect GRILLO training, concurrent rehabilitation, spontaneous recovery, regression to the mean, or a combination of these factors.

#### 3.3.3. Tinetti Balance Scale

Paired observations were available for 22 patients. Median Tinetti Balance Scale scores increased from 1 (IQR 0–2) at baseline to 2 (IQR 1–5) post-intervention, corresponding to a median change of +1 point (IQR 0–3), with a statistically significant pre–post difference (*p* = 0.006; effect size r = 0.59).

Thirteen out of 22 (59%) improved, 8 (36%) remained stable, and 1 (5%) showed decline. Despite the statistically significant change, absolute scores remained low, reflecting the severity of baseline impairment and the presence of floor effects. A proportion of patients transitioned from a score of 0 at baseline to a non-zero score post-intervention, indicating the emergence of measurable balance ability in individuals who were initially unable to perform the task.

#### 3.3.4. Tinetti Gait-Related Items

Paired observations were available for 20 patients. At baseline, the majority of patients (median score 0, IQR 0–0) were unable to perform gait-related items, resulting in marked floor effects. Following the intervention period, the median remained 0 (IQR 0–3), and no formal inferential analysis was performed because of the limited distribution of scores and the persistence of floor effects.

Six patients (30%) showed improvement, 13 (65%) remained at baseline levels, and 1 (5%) showed decline. Given the extent of baseline floor effects and the small absolute changes, these data are presented descriptively and interpreted with caution. The emergence of measurable gait scores in a subset of patients may indicate clinically relevant functional transitions, but the magnitude of change does not support strong conclusions about walking recovery in this population.

#### 3.3.5. Patient-Reported Perception

**Figure 7:**
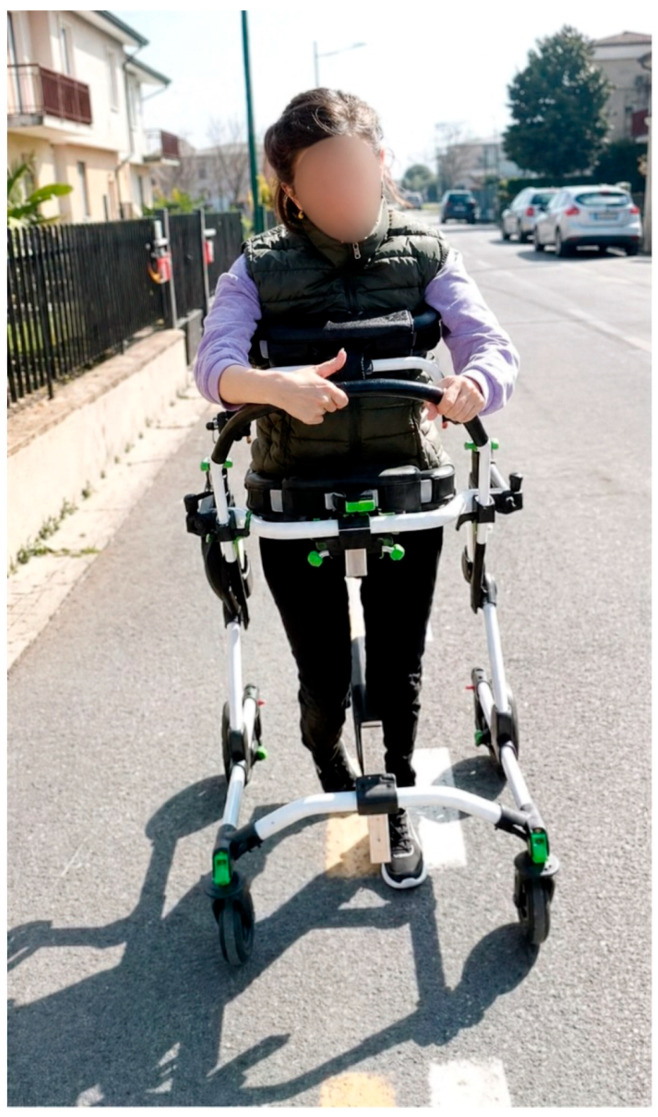
Example of GRILLO use in a real-world setting after discharge, illustrating device configuration and support strategies during daily functional activities.

Review of clinical documentation revealed that among patients for whom perceptions were recorded, most reported that the device was perceived as useful during rehabilitation. However, these reports were extracted from routine notes and were not collected through a validated satisfaction or usability instrument. Examination of discharge records indicated that only one patient received a prescription for continued device use after discharge. This discrepancy suggests that perceived clinical usefulness during supervised training does not necessarily translate into sustained use outside the rehabilitation environment, possibly because of practical and logistical barriers related to device size, space requirements, caregiver support, and everyday manageability. Nevertheless, when continued use after discharge is feasible, GRILLO may support autonomy in daily activities, as illustrated in Figure 7.

## 4. Discussion

This retrospective observational study examined the use of a modular gait trainer in a heterogeneous cohort of patients with severe neurological motor impairment undergoing inpatient rehabilitation. The main findings indicate that GRILLO-based training could be implemented in selected patients within routine clinical practice. No serious device-related adverse events were documented in available clinical records, but adverse events were not monitored prospectively or systematically. Exploratory pre–post functional changes were observed across measures of independence, trunk control, and balance, although individual responses were highly variable and no causal inference regarding device-specific efficacy can be made. Feasibility should be interpreted by considering both completion and non-completion. Although most patients in the paired functional-analysis cohort completed the planned sessions, five patients interrupted training because of pain or poor tolerance to prolonged upright positioning. These cases suggest that initial clinical indication alone is not sufficient to determine suitability for GRILLO-based training. The timing of introduction, tolerance to sustained upright effort, pain or discomfort, fatigue, postural control, behavioral engagement, and medical stability are central feasibility determinants. Future prospective studies should define feasibility a priori, including completion rate, reasons for discontinuation, tolerance thresholds, adverse symptoms, therapist burden, and patient/caregiver acceptability.

### 4.1. Principal Findings

Among the assessed domains, trunk control showed the most consistent improvement. This observation is clinically plausible, as trunk stabilization is a prerequisite for more complex motor functions, including transfers, standing, and gait [6,20,21]. The median improvement on the Trunk Control Test was 12 points, and 71% of patients with paired TCT data demonstrated gains, with no patients showing decline. These findings indicate that trunk control was the domain with the clearest change in this cohort. However, the uncontrolled retrospective design prevents attribution of this change specifically to GRILLO. All patients received standard physical therapy and other rehabilitative interventions alongside GRILLO sessions, and exposure to these concurrent treatments was not quantified in a standardized way. Improvements may therefore reflect GRILLO-based training, concurrent rehabilitation, spontaneous recovery, regression to the mean, repeated testing, or combined effects. Accordingly, this finding should be interpreted as exploratory and hypothesis-generating rather than as evidence of device-specific efficacy.

Changes in functional independence, as measured by the Barthel Index, were more modest and highly variable. The median increase of 3 points is below commonly cited thresholds for minimal clinically important difference (typically estimated at 10–15 points in stroke populations) [19]. However, the distribution of individual changes indicates that while the group median was limited, a meaningful subset of patients (61% showing improvement) experienced gains, with some demonstrating changes exceeding 10 points. This pattern suggests that the intervention may be more effective in selected individuals rather than uniformly beneficial across a broad population.

Balance outcomes were characterized by limited absolute changes and persistent floor effects. The median improvement of 1 point on the Tinetti Balance Scale, while statistically significant, is of uncertain clinical relevance given the ordinal nature of the scale and the severity of baseline impairment. The transition from a score of 0 to a non-zero score in some patients may represent an early functional threshold crossing, but interpretation is constrained by the limited sensitivity of the measure in this population.

The gait-related findings further underscore the limitations of standard outcome measures in severely impaired populations. Most patients remained unable to perform gait-related items at baseline, and floor effects persisted at follow-up. Although a subset of patients showed measurable improvement, the magnitude of change was small and does not support strong conclusions regarding walking recovery. In this cohort, gait-related data are more useful as descriptive indicators of emerging capacity than as robust efficacy outcomes.

### 4.2. Heterogeneity and Patient Selection

An important aspect of this study is the substantial heterogeneity of the cohort. The variability in time from neurological event to enrollment (median 196 days, range 41 to 1888 days) indicates that patients were distributed across subacute and chronic phases of recovery. This variability likely influenced both tolerance to the intervention and responsiveness to training.

In earlier phases, spontaneous neurological recovery may contribute more substantially to improvement [2,6], whereas in later phases compensatory strategies and longer-standing impairments are likely to play a larger role. This variability reinforces the view that patient selection is central to the interpretation of results. Some patients appeared to derive measurable benefit, whereas others showed little change, suggesting that this type of intervention is unlikely to have the same relevance across all severe neurological presentations.

The retrospective nature of this study prevented systematic characterization of factors predicting response. Future research should focus on identifying baseline characteristics, impairment profiles, or training parameters that distinguish responders from non-responders. Such work would inform more targeted patient selection and potentially improve the efficiency of resource allocation in clinical practice.

### 4.3. Implementation Considerations

From a practical perspective, this study highlights a potential gap between selected in-clinic feasibility and broader real-world implementation. Although the device was generally perceived as useful during supervised sessions, review of discharge records revealed that only one patient received prescriptions for continued use after discharge.

This discrepancy likely reflects multiple factors. First, the device requires a certain level of caregiver capacity and environmental space that may not be available in all home settings. Second, the cost of acquisition or rental may represent a barrier for many families. Third, the progressive nature of rehabilitation means that patients who benefited from GRILLO support during more impaired phases may transition to less-supported mobility aids as recovery progresses, reducing the perceived need for continued device use.

These considerations are relevant when evaluating the overall clinical impact and cost-effectiveness of such devices. While GRILLO training may serve an important role as a transitional intervention during intensive inpatient rehabilitation, its utility may be primarily as a therapeutic tool rather than as a long-term assistive device for most users. However, in selected cases, continued use after discharge may support autonomy and participation in daily activities, suggesting that the potential long-term value of the device should not be dismissed. This is particularly relevant for patients with traumatic brain injury and other acquired brain injuries, in whom motor disability may persist as part of a chronic condition. This consideration aligns with the growing recognition of traumatic brain injury as a chronic health condition rather than a time-limited event [25,26]. Recent evidence indicates that individuals with TBI experience long-term health consequences, including reduced life expectancy and increased risk of chronic comorbidities, extending well beyond the acute and subacute phases of recovery. A recent meta-analysis reported persistent reductions in survival and increased long-term mortality risk following TBI [27], reinforcing the need to conceptualize rehabilitation within a chronic care framework. Similarly, systematic reviews have emphasized the enduring and multidimensional impact of TBI across functional, medical, and psychosocial domains [28,29]. Within this perspective, interventions targeting postural control and mobility, even when applied in later stages, may contribute to maintaining functional capacity and preventing secondary complications, rather than solely promoting short-term recovery.

Longer follow-up studies are therefore needed to determine whether and for whom GRILLO use can translate into sustained functional gains beyond the inpatient rehabilitation setting. These findings also highlight the need for further development of assistive technologies that preserve adequate postural support while improving portability, usability, and integration into domestic environments.

### 4.4. Comparison with Literature

These findings are broadly consistent with the literature suggesting that assisted verticalization and supported gait-related training may have a role in neurological rehabilitation [7,8,9,10,11,15,30]. At the same time, the available literature remains heterogeneous and does not provide strong evidence for a single device or protocol in severely impaired, diagnostically mixed sABI populations. Robotic verticalization systems, body-weight-supported treadmill training, and robot-assisted gait devices differ substantially from GRILLO in terms of unloading mechanism, therapist involvement, degree of active participation, set-up complexity, and target population [12,13,14,15,30]. Compared with more technology-intensive systems, GRILLO may offer a clinically flexible form of supported upright activity, but this potential advantage remains qualitative and must be tested in prospective comparative studies.

Our results add to this literature by describing the use of a modular gait trainer in routine clinical practice within a highly heterogeneous inpatient cohort. In this context, even modest improvements in trunk control or the emergence of measurable balance or gait-related performance may represent meaningful steps within a broader rehabilitation process.

### 4.5. Limitations

Several limitations should be considered when interpreting these findings. The retrospective observational design, combined with the absence of a control group, does not allow any causal inference regarding the specific contribution of GRILLO-based training. The functional changes observed may reflect the intervention itself, concurrent multidisciplinary rehabilitation, spontaneous recovery, or a combination of these factors.

The sample was small and diagnostically heterogeneous. Although this reflects real-world rehabilitation practice, it also increases variability, reduces the precision of estimates, and limits conclusions about specific clinical subgroups. Inclusion in the paired functional analysis required both completion of at least 10 GRILLO sessions and availability of paired assessments, which may have introduced survivorship and selection bias. Patients who discontinued training because of pain, poor tolerance to prolonged upright positioning, poor compliance or motivation, or an incomplete clinical pathway remain highly relevant to the interpretation of feasibility. Feasibility in an unselected clinical population may therefore be lower than suggested by completion rates calculated only among those who completed the treatment. In addition, outcome assessments were not standardized prospectively for research purposes, and assessors were not blinded. Because data were derived from routine clinical documentation, variability in assessment timing, completeness, and consistency of measurement may have affected the results. Finally, the available retrospective records did not support a valid comparison of the rate of change before and during the GRILLO period, a reliable dose–response analysis, or comparison with a historical non-GRILLO cohort. These analyses would have required more standardized assessment timing, documentation procedures, and quantification of concurrent rehabilitation exposure than were available in the present dataset.

Safety data should also be interpreted cautiously. Serious adverse events attributable to the device were not identified in the clinical records, but minor events were not captured through a structured monitoring procedure and may therefore be underreported. Follow-up was limited to the end of the intervention period, so the durability of any observed changes remains unknown. Finally, some of the outcome measures used in routine practice appeared only partially sensitive to early changes in this severely impaired population, particularly for balance and gait, where floor effects were prominent. More targeted measures may be needed in future studies to better characterize clinically relevant change.

### 4.6. Future Directions

Despite these limitations, the study provides clinically relevant information on the use of a modular gait trainer in a population that is often underrepresented in controlled research. The findings support the feasibility of integrating this approach into routine inpatient multidisciplinary rehabilitation and suggest that selected patients may derive functional benefit also in the domestic or ecological environment.

Further research should use prospective controlled designs capable of clarifying the specific contribution of the device beyond standard care. Future studies should define a priori feasibility endpoints, systematically monitor adverse events, quantify concurrent rehabilitation exposure, and use appropriate comparator groups such as matched historical cohorts, usual care, or alternative supported verticalization/gait-training devices. Outcome measures should be selected to capture early changes in severely impaired patients, including postural control, tolerance to upright posture, effort, assistance level, movement quality, discharge destination, caregiver burden, and participation in daily activities. Longer follow-up is needed to establish whether any gains are sustained and whether they translate into reduced assistance needs or meaningful functional outcomes. Better definition of patient selection criteria and response predictors would also help guide clinical decision-making.

## 5. Conclusions

This retrospective study suggests that GRILLO-based training can be implemented in selected adults with severe acquired brain injury within a supervised inpatient rehabilitation setting. However, feasibility should be interpreted cautiously because patients who interrupted training or did not complete the minimum exposure were not included in the paired functional-analysis cohort.

No serious device-related adverse events were documented in available clinical records, but adverse events were not monitored prospectively or systematically. Exploratory pre–post changes were observed in trunk control, balance, and activities of daily living, with substantial individual variability and floor effects in balance and gait-related measures.

Because all patients received concurrent multidisciplinary rehabilitation and no comparator group was available, these changes cannot be attributed specifically to GRILLO. The observed changes may reflect GRILLO training, other therapeutic interventions, spontaneous recovery, regression to the mean, repeated testing, or combined effects. Practical limitations related to device configuration, supervision requirements, caregiver support, and applicability beyond supervised clinical settings should be considered when interpreting the potential clinical role of this technology.

These preliminary findings support further prospective controlled investigation rather than conclusions about efficacy. Future studies should include appropriate comparison groups, standardized adverse-event monitoring, predefined feasibility endpoints, longer follow-up, clearer patient selection criteria, and more sensitive outcome measures to determine device-specific efficacy and identify the patient populations most likely to benefit.

## Figures and Tables

**Table 1:** Baseline demographic and clinical characteristics of the study cohort. The table describes the paired functional-analysis cohort (n = 22). Percentages may not sum to 100% because of rounding.

Characteristic	Value
Patients included in paired functional-analysis cohort, n	22
Female sex, n (%)	10 (45%)
Age, years, median (IQR)	53 (39–58)
Age range, years	20–75
Neurological diagnosis, n (%)	
Traumatic brain injury	8 (36%)
Hemorrhagic stroke or subarachnoid hemorrhage	6 (27%)
Ischemic stroke	3 (14%)
Anoxic encephalopathy	2 (9%)
Other acquired neurological conditions	3 (14%)
Time from neurological event to GRILLO training	
Days, median (IQR)	196 (116–360)
Range, days	41–1888
Subacute phase, ≤6 months, n (%)	10 (45%)
Chronic phase, >6 months, n (%)	12 (55%)
GRILLO training completion in paired functional-analysis cohort	
15 sessions completed, n (%)	20 (91%)
10 sessions completed, n (%)	2 (9%)

Abbreviation: IQR, interquartile range.

**Table 2:** Pre- and post-intervention functional outcomes.

Outcome Measure	N	Baseline Score, Median (IQR)	Post-Intervention Score, Median (IQR)	Change Score, Median (IQR)	*p* Value	Effect Size, r
Barthel Index	20	16 (9.75–26.25)	22.5 (14.5–35.5)	+3 (0–12.25)	0.008	0.59
Trunk Control Test	21	72 (37–74)	74 (49–87)	+12 (0–13)	<0.001	0.77
Tinetti Balance Scale	22	1 (0–2)	2 (1–5)	+1 (0–3)	0.006	0.59
Tinetti Gait subscale	20	0 (0–0)	0 (0–3)	0 (descriptive)	Not tested	Not calculated

## Data Availability

The data presented in this study are available on reasonable request from the corresponding author due to privacy and ethical reasons.

## Abbreviations

The following abbreviations are used in this manuscript:

ABI	Acquired Brain Injury
TBI	Traumatic Brain Injury
BI	Barthel Index
IQR	Interquartile Range
POMA	Performance-Oriented Mobility Assessment
TCT	Trunk Control Test
